# Supplementary material for: Theoretical Insight into Psittacofulvins and Their Derivatives
Source: Molecules. 2024 Jun 10;29(12):2760. doi: 10.3390/molecules29122760 (PMC11206620; doi:10.3390/molecules29122760)
Supplement: Supplementary file 1 [file molecules-29-02760-s001.zip › molecules-3021636-supplementary.pdf]

Supporting Information

Theoretical Insight into Psittacofulvins and their Derivatives

Marcin Molski

Quantum Chemistry Department
Faculty of Chemistry
Adam Mickiewicz University
ul. Uniwersytetu Poznańskiego 8, 61-614 Poznań, Poland
mamolski@amu.edu.pl

1. Thermodynamic Descriptors and Scavenging Mechanisms

Free radicals can be deactivated by the R─H compound via the three fundamental mechanisms specified below.

HAT (*Hydrogen Atom Transfer*)

$$R{-}H + X^{\bullet} \rightarrow R^{\bullet} + X{-}H$$

This mechanism is widely applied in processes in which the hydrogen atom is transferred from the basic compound to the reactive intermediate. The antiradical potency of a compound is related to the low bond dissociation enthalpy (BDE) of the N–H bond in amines, S–H in organosulfur compounds, O–H in polyphenols, alcohols, carboxylic acids and O=C–H in aldehydes. Hence, crucial for calculating the BDE parameter is reaction

$$R{-}H \rightarrow R^{\bullet} + H^{\bullet}$$

initiating the HAT process, which allows defining the BDE parameter [20]

$$BDE = H(R^{\bullet}) + H(H^{\bullet}) - H(R-H) \qquad \text{(S1)}$$

in which $H(R^{\bullet})$, $H(H^{\bullet})$ and H (R─H) denote enthalpies of the radical, hydrogen atom, and the neutral molecule.

SET-PT (*Single Electron Transfer Followed by Proton Transfer*)

$$R{-}H + X^{\bullet} \rightarrow R{-}H^{\bullet+} + X^{-}$$

$$R{-}H^{\bullet+} \rightarrow R^{\bullet} + H^{+}$$

This scenario involves the transfer of an electron from the parent compound

$$R{-}H \rightarrow R{-}H^{\bullet+} + e^{-}$$

generating a cation radical $R{-}H^{\bullet+}$ and, in the next step, the proton transfer from the cation radical producing parent agent in the radical form $R^{\bullet}$. These two stages are characterized by the adiabatic ionization potential (AIP) and the proton dissociation enthalpy (PDE) calculated from the formulae [20]

$$\begin{aligned} AIP &= H(R-H^{\bullet+}) + H(e^{-}) - H(R-H) \\ PDE &= H(R^{\bullet}) + H(H^{+}) - H(R-H^{\bullet+}) \end{aligned} \qquad \text{(S2)}$$

Here, $H(R{-}H^{\bullet+})$, $H(e^{-})$, $H(H^{+})$, and H(R─H) represent the enthalpies of the cation, electron, proton, and the parent compound, respectively. AIP is determined using the enthalpies of the optimized cationic state and the optimized neutral molecule. The Vertical Ionization Potential (VIP) defined as the difference between the energy of the optimized neutral structure and that of the corresponding cationic system at the neutral geometry is not considered in this work.

SPLET (*Sequential Proton Loss Electron Transfer*)

$$R\text{—}H \rightarrow R^- + H^+$$
$$R^- + X^{\bullet} + H^+ \rightarrow R^{\bullet} + X\text{—}H$$

The second step of the reaction above specified is initiated by the reaction

$$R^- \rightarrow R^{\bullet} + e^-$$

The SPLET process is therefore characterized by the proton affinity (PA) and electron transfer enthalpy (ETE) calculated according to the formulae [20]

$$PA = H(R^-) + H(H^+) - H(R-H)$$
$$ETE = H(R^{\bullet}) + H(e^-) - H(R^-) \quad \text{(S3)}$$

If the reactions specified above are not sequential and involve the concerted shift of a single electron and a single proton they are called Concerted Proton-Electron Transfer (CPET). This pathway belongs to the wide class of a Proton-Coupled Electron Transfer (PCET), which involves the shift of electrons and protons from one molecule (atom) to another.

TMC (*Transition Metals Chelation*)

This mechanism is related to the chelation of the transition metal ions (especially Fe^{2+} and Cu^{2+}), which produces stable complexes that remove them from the reaction medium and contribute to the slowing down of the radical cascade reactions. Vital for this mechanism is the dissociation decay [34]

R—H → R- + H+

The parameter characterizing TMC in solvents is the free Gibbs acidity [34]

$$G_{acidity} = G(R^-) - G(R-H) \quad \text{(S4)}$$

in which $G(R^-)$ and G(R—H) denote the Gibbs free energy of the anion and its parent compound, respectively. A lower value of this parameter indicates a greater ability to chelate metals and, consequently, a greater potency to slow down radical processes.

RAF (*Radical Adduct Formation*)

In this scenario, radicals are deactivated in the reaction

$$R\text{—}H + X^{\bullet} \rightarrow R\text{—}XH^{\bullet}$$

which is characterized by the enthalpy or Gibbs free energies formation calculated for a given radical and an antioxidant considered [35]. Hence, this pathway is characterized by the descriptor depending on the radical type. Consequently, it will not be considered here.

In calculations of the BDE, PDE, AIP, ETE, PA, $G_{acidity}$ parameters, we used the values of the electron, proton and hydrogen enthalpies in the gas phase, water and benzene recommended by Rimarčik et al. [20].

2. Chemical Activity Descriptors

An important source of information on the reactivity of chemical compounds is the difference in the energies of the HOMO and LUMO frontier orbitals. The shape of these orbitals, as well the energy gap (usually expressed in electron volts [eV])

$$\Delta E = E_{LUMO} - E_{HOMO} \tag{S5}$$

have an impact on the chemical reactivity of compounds. A large energy difference defines a *hard* molecule that is more stable and less active, while a small energy gap defines a *soft* molecule that is less stable and more reactive. Based on the energy of the LUMO and HOMO orbitals, as well as the Koopmans' theorem for the closed shell molecules, one may define the global activity descriptors [36,37], which model the physicochemical properties of chemical compounds [38]. The most important chemical activity descriptors are specified below.

Ionization potential

$$IP = -E_{HOMO} \tag{S6}$$

It describes the minimum energy required to remove an electron from the molecule's HOMO orbital and move it to infinity. The small IP value indicates a greater tendency of the molecule to participate in the chemical reaction related to electron transfer. The best radical scavengers are endowed with low IP values.

Electron affinity

$$EA = -E_{LUMO} \tag{S7}$$

It expresses the ability of a molecule to accept an electron and produce an anion. Due to the fact that radicals scavenging might act either via donating or accepting electrons, the EA descriptor is useful to characterize the capacity of a compound to accept electrons.

Chemical hardness

$$\eta \approx \frac{E_{LUMO} - E_{HOMO}}{2} \tag{S8}$$

It describes a molecule with a low susceptibility to deformation or polarization of the electron cloud under the influence of external factors, e.g., reagents.

Chemical softness

$$S \approx \frac{1}{E_{LUMO} - E_{HOMO}} \tag{S9}$$

The inverse of chemical hardness – the S-descriptor characterizes molecules with the high susceptibility to deformation and polarization of the electron cloud.

Chemical potential

$$\mu \approx \frac{E_{LUMO} + E_{HOMO}}{2} \tag{S10}$$

Employed to describe the thermodynamic activity of substances and used, for example, in the derivation of the phase equilibrium constants.

Electronegativity

$$\chi = -\mu \approx -\frac{E_{LUMO} + E_{HOMO}}{2} = \frac{EA + IP}{2} \tag{S11}$$

It characterizes a tendency to attract electrons that create the chemical bond. The common electron pair is shifted towards the atom having a high value of χ, which is accompanied by the formation of a polar or ionic bond.

Electrophilicity Index

$$\omega \approx \frac{\chi^2}{2\eta} = \frac{\left(E_{LUMO} + E_{HOMO}\right)^2}{4\left(E_{LUMO} - E_{HOMO}\right)} \tag{S12}$$

This descriptor was introduced by Robert Parr and coworkers [39]. It measures the energy change of an electrophilic reagent (acceptor) when it is saturated with electrons provided by another reagent (donor). Higher values of ω characterize a strong electrophile, whereas a strong nucleophile is described by lower values of ω.

Electro-donating and electro-accepting power

A radical scavenger (RS) can act in two ways: either by donating electrons to, or accepting electrons from a radical (R)

$$RS + R \rightarrow RS^{+} + R^{-} \qquad RS + R \rightarrow RS^{-} + R^{+}$$

Following this fact, Gázquez et al. [40] introduced new descriptors characterizing the electro-donating ω^- and electro-accepting ω^+ power of RS, defined in the following manner

$$\omega^- = \frac{\left(3IP + EA\right)^2}{16\left(IP - EA\right)} \qquad \omega^+ = \frac{\left(IP + 3EA\right)^2}{16\left(IP - EA\right)} \tag{S13}$$

To characterize the relative electro-accepting (donating) power of an arbitrary radical scavenger X, Martinez [11] introduced the acceptance Ra and donation Rd indexes

$$Ra = \frac{\omega_X^+}{\omega_F^+} \qquad Rd = \frac{\omega_X^-}{\omega_{Na}^-} \tag{S14}$$

defined by the IP and EA for the F and Na atoms. They represent a good electron acceptor (F) and a good donor (Na), respectively. In the calculations, the well-known experimental values for F [11]: IP = 17.42282, EA = 3.4011898 [eV], and for Na: IP = 5.1391, EA = 0.547926 [eV] are employed. The descriptors Ra and Rd are useful for classifying any compound X in terms of its electron donating (accepting) capacity. Hence, Ra and Rd can be applied to characterize the antiradical capacity of X in terms of its electro-donating (accepting) power with respect to the F and Na atoms, taken as the reference points [11].

Table S1. A comparison of the chemical activity descriptors [eV] of the natural P6-9 and projected P4, P5, P10 psittacofulvins, determined at the DFT/B3LYP, M06-2X, BHandHLYP/QZVP theory levels in the water medium, using the C-PCM solvation model. The descriptors are calculated from the energies $E_{LUMO} = –EA$, $E_{HOMO} = –IP$. The energy values E of the compounds considered are presented.

Descriptor	P4	P5	P6	P7	P8	P9	P10
				B3LYP			
E[a]	-463.684180	-541.127309	-618.570497	-696.013717	-773.456958	-850.900213	-928.343478
EA	2.7141	2.8229	2.9029	2.9633	3.0112	3.0488	3.0792
IP	5.9095	5.6611	5.4771	5.3342	5.2235	5.1334	5.0572
ΔE	3.1954	2.8381	2.5742	2.3709	2.2123	2.0847	1.9779
η	1.5977	1.4191	1.2871	1.1855	1.1061	1.0423	0.9889
S	0.3129	0.3523	0.3885	0.4218	0.4520	0.4797	0.5056
χ=-μ	4.3118	4.2420	4.1900	4.1488	4.1174	4.0911	4.0682
ω	5.8181	6.3402	6.8201	7.2598	7.6629	8.0287	8.3673
ω^+	3.8620	4.3966	4.8859	5.3336	5.7425	6.1134	6.4568
ω^-	8.1737	8.6386	9.0760	9.4824	9.8599	10.2045	10.5251
Ra[b]	1.1352	1.2924	1.4362	1.5678	1.6880	1.7970	1.8980
Rd[b]	2.3557	2.4896	2.6157	2.7328	2.8416	2.9409	3.0333
				M06-2X			
E[a]	-463.461246	-540.867317	-618.273451	-695.679623	-773.085820	-850.492033	-927.898256
EA	1.7402	1.8792	1.9775	2.0493	2.1029	2.1440	2.1761
IP	7.0954	6.8222	6.6238	6.4747	6.3593	6.2681	6.1947
ΔE	5.3552	4.9429	4.6463	4.4254	4.2564	4.1242	4.0186
η	2.6776	2.4715	2.3232	2.2127	2.1282	2.0621	2.0093
S	0.1867	0.2023	0.2152	0.2260	0.2349	0.2425	0.2488
χ=-μ	4.4178	4.3507	4.3006	4.2620	4.2311	4.2060	4.1854
ω	3.6444	3.8294	3.9806	4.1046	4.2059	4.2896	4.3591
ω^+	1.7703	1.9630	2.1207	2.2502	2.3564	2.4443	2.5176
ω^-	6.1880	6.3137	6.4213	6.5122	6.5875	6.6504	6.7030
Ra[b]	0.5204	0.5770	0.6234	0.6614	0.6927	0.7185	0.7400
Rd[b]	1.7834	1.8196	1.8506	1.8768	1.8985	1.9166	1.9318
				BHandHLYP			
E[a]	-463,378821	-540.769203	-618.199634	-695.550097	-772.940581	-850.331080	-927.721589
EA	1.4604	1.5984	1.6955	1.7663	1.8191	1.8588	1.8901
IP	6.7732	6.4875	6.2798	6.1231	6.0017	5.9060	5.8284
ΔE	5.3127	4.8891	4.5843	4.3568	4.1827	4.0471	3.9383
η	2.6564	2.4445	2.2922	2.1784	2.0913	2.0236	1.9692
S	0.1882	0.2045	0.2181	0.2295	0.2391	0.2471	0.2539
χ=-μ	4.1168	4.0429	3.9877	3.9447	3.9104	3.8824	3.7818
ω	3.1901	3.3432	3.4687	3.5716	3.6559	3.7243	3.7818
ω^+	1.4637	1.6273	1.7614	1.8715	1.9621	2.0361	2.0983
ω^-	5.5805	5.6703	5.7491	5.8162	5.8725	5.9185	5.9576
Ra[b]	0.4303	0.4784	0.5178	0.5501	0.5768	0.5985	0.6168
Rd[b]	1.6083	1.6342	1.6569	1.6762	1.6925	1.7057	1.7170

[a] Energy in Hartree unit [Ha]. [b] Dimensionless parameter.

Table S2. A comparison of the chemical activity descriptors [eV] of the carboxylic derivatives POOH6−9 of psittacofulvins P6−9 determined at the DFT/B3LYP, M06-2X, BHandHLYP/QZVP theory levels in the water medium, using the C-PCM solvation model. The descriptors are calculated from the energies E_{LUMO} = –EA, E_{HOMO} = –IP. The energy values E of the compounds considered are presented.

Descriptor	POOH6	POOH7	POOH8	POOH9
		B3LYP		
E[a]	–693.861568	–771.304755	–848.747974	–926.191214
EA	2.7856	2.8572	2.9133	2.9581
IP	5.4453	5.3073	5.1987	5.1108
ΔE	2.6596	2.4501	2.2855	2.1527
η	1.3298	1.2251	1.1427	1.0763
S	0.3760	0.4081	0.4375	0.4645
χ=-μ	4.1155	4.0823	4.0560	4.0345
ω	6.3681	6.8016	7.1981	7.5613
ω^+	4.4766	4.9136	5.3129	5.6786
ω^-	8.5921	8.9959	9.3689	9.7131
Ra[b]	1.3159	1.4444	1.5617	1.6692
Rd[b]	2.4762	2.5926	2.7001	2.7993
		M06-2X		
E[a]	–693.544248	–770.950421	–848.356619	–925.762833
EA	1.8735	1.9565	2.0194	2.0681
IP	6.6101	6.4624	6.3487	6.2586
ΔE	4.7367	4.5059	4.3293	4.1906
η	2.3683	2.2530	2.1647	2.0953
S	0.2111	0.2219	0.2310	0.2386
χ=-μ	4.2418	4.2095	4.1840	4.1633
ω	3.7987	3.9325	4.0436	4.1363
ω^+	1.7913	2.1094	2.2222	2.3165
ω^-	6.2157	6.3189	6.4062	6.4799
Ra[b]	0.5802	0.6201	0.6532	0.6808
Rd[b]	1.7914	1.8211	1.8463	1.8675
		BHandHLYP		
E[a]	–693.416574	–770.807034	–848.197518	–925.588018
EA	1.5891	1.6705	1.7320	1.7794
IP	6.2649	6.1100	5.9903	5.8956
ΔE	4.6757	4.4395	4.2583	4.1163
η	2.3379	2.2198	2.1292	2.0581
S	0.2139	0.2252	0.2348	0.2429
χ=-μ	3.9270	3.8903	3.8612	3.8375
ω	3.2982	3.4090	3.5010	3.5776
ω^+	1.6269	1.7413	1.8366	1.9161
ω^-	5.5539	5.6316	5.6978	5.7536
Ra[b]	0.4782	0.5119	0.5399	0.5632
Rd[b]	1.6006	1.6230	1.6421	1.6582

[a] Energy in Hartree unit [Ha]. [b] Dimensionless parameter.

Table S3. A comparison of the chemical activity descriptors [eV] of the dialdehyde derivatives D6–9 of psittacofulvins P6–9 determined at the DFT/B3LYP, M06-2X, BHandHLYP/QZVP theory levels in the water medium, using the C-PCM solvation model. The descriptors are calculated from the energies $E_{LUMO} = -EA$, $E_{HOMO} = -IP$. The energy values E of the compounds considered are presented.

Descriptor	D6	D7	D8	D9
		B3LYP		
E[a]	–692.620804	–770.064694	–847.508432	–924.952064
EA	3.2975	3.3007	3.3032	3.3051
IP	5.8379	5.6510	5.5019	5.3802
ΔE	2.5405	2.3502	2.1987	2.0751
η	1.2702	1.1751	1.0993	1.0376
S	0.3936	0.4255	0.4548	0.4819
χ=-μ	4.5677	4.4759	4.4025	4.3427
ω	8.2127	8.5239	8.8154	9.0879
ω^+	6.0876	6.4329	6.7516	7.0463
ω^-	10.6553	10.9087	11.1541	11.3890
Ra[b]	1.7894	1.8909	1.9846	2.0712
Rd[b]	3.0709	3.1439	3.2146	3.2823
		M06-2X		
E[a]	–692.302716	–769.709285	–847.115740	–924.522120
EA	2.3228	2.3345	2.3464	2.3519
IP	6.9441	6.7462	6.5890	6.4649
ΔE	4.6213	4.4118	4.2425	4.1130
η	2.3107	2.2059	2.1213	2.0565
S	0.2164	0.2267	0.2357	0.2431
χ=-μ	4.6334	4.5404	4.4677	4.4004
ω	4.6456	4.6727	4.7048	4.7250
ω^+	2.6177	2.6782	2.7361	2.7778
ω^-	7.2511	7.2186	7.2038	7.1862
Ra[b]	0.7695	0.7873	0.8043	0.8165
Rd[b]	2.0898	2.0804	2.0761	2.0711
		BHandHLYP		
E[a]	–692.177317	–769.568274	–846.959087	–924.349805
EA	2.0662	2.0749	2.0803	2.0844
IP	6.6167	6.4107	6.2483	6.1174
ΔE	4.5506	4.3359	4.1680	4.0330
η	2.2753	2.1679	2.0803	2.0165
S	0.2198	0.2306	0.2399	0.2480
χ=-μ	4.3414	4.2428	4.1643	4.1009
ω	4.1419	4.1517	4.1606	4.1699
ω^+	2.2556	2.3013	2.3390	2.3715
ω^-	6.5971	6.5441	6.5033	6.4724
Ra[b]	0.6630	0.6765	0.6875	0.6971
Rd[b]	1.9013	1.8860	1.8742	1.8654

[a] Energy in Hartree unit [Ha]. [b] Dimensionless parameter.
